# Maternal Melatonin Therapy Attenuates Methyl-Donor Diet-Induced Programmed Hypertension in Male Adult Rat Offspring

**DOI:** 10.3390/nu10101407

**Published:** 2018-10-02

**Authors:** You-Lin Tain, Julie Y. H. Chan, Chien-Te Lee, Chien-Ning Hsu

**Affiliations:** 1 Department of Pediatrics, Kaohsiung Chang Gung Memorial Hospital and Chang Gung University, College of Medicine, Kaohsiung 833, Taiwan; tainyl@hotmail.com; 2 Institute for Translational Research in Biomedicine, Kaohsiung Chang Gung Memorial Hospital and Chang Gung University, College of Medicine, Kaohsiung 833, Taiwan; jchan@cgmh.org.tw; 3 Division of Nephrology, Kaohsiung Chang Gung Memorial Hospital and Chang Gung University, College of Medicine, Kaohsiung 833, Taiwan; ctlee33@cgmh.org.tw; 4 Department of Pharmacy, Kaohsiung Chang Gung Memorial Hospital, Kaohsiung 833, Taiwan; 5 School of Pharmacy, Kaohsiung Medical University, Kaohsiung 807, Taiwan

**Keywords:** developmental origins of adult health and disease (DOHaD), hypertension, melatonin, methylation, nutrient-sensing signal, oxidative stress

## Abstract

Although pregnant women are advised to consume methyl-donor food, some reports suggest an adverse outcome. We investigated whether maternal melatonin therapy can prevent hypertension induced by a high methyl-donor diet. Female Sprague-Dawley rats received either a normal diet, a methyl-deficient diet (L-MD), or a high methyl-donor diet (H-MD) during gestation and lactation. Male offspring were assigned to four groups (*n* = 7–8/group): control, L-MD, H-MD, and H-MD rats were given melatonin (100 mg/L) with their drinking water throughout the period of pregnancy and lactation (H-MD+M). At 12 weeks of age, male offspring exposed to a L-MD or a H-MD diet developed programmed hypertension. Maternal melatonin therapy attenuated high methyl-donor diet-induced programmed hypertension. A maternal L-MD diet and H-MD diet caused respectively 938 and 806 renal transcripts to be modified in adult offspring. The protective effects of melatonin against programmed hypertension relate to reduced oxidative stress, increased urinary NO_2_^−^ level, and reduced renal expression of sodium transporters. A H-MD or L-MD diet may upset the balance of methylation status, leading to alterations of renal transcriptome and programmed hypertension. A better understanding of reprogramming effects of melatonin might aid in developing a therapeutic strategy for the prevention of hypertension in adult offspring exposed to an excessive maternal methyl-supplemented diet.

## 1. Introduction

Hypertension remains a leading threat to human health. An estimated one in three adults worldwide have high blood pressure (BP). Adulthood hypertension can originate in early life. Maternal nutrition plays a critical role in fetal growth and development. Suboptimal nutrition in fetal and infantile life induces morphological changes and functional adaption, increasing the risk for developing chronic diseases in adulthood by so-called nutritional programming [1]. This concept is formerly known as the developmental origins of adult health and disease (DOHaD) [2].

Pregnant women are currently advised to consume methyl-donor food to reduce adverse birth outcomes, such as neural tube defects [3]. Such methyl-donor nutrients include molecules that directly provide methyl donors or serve as cofactors, such as folic acid, methionine, choline, and vitamins B2, B6, and B12 [4]. Methylation is a process that involves the addition of a methyl group to DNA, protein, or other molecules. DNA methylation, catalyzed by the DNA methyltransferases (DNMTs), is a key player in epigenetic silencing of transcription and is essential for normal fetal development [5]. Global DNA hypomethylation has been shown to be associated with many diseases [6]. On the other hand, protein arginine methylation is performed by protein arginine methyltransferases (PRMTs), which methylate protein-incorporated arginine residues to generate symmetric dimethylarginine (SDMA), or asymmetric dimethylarginine (ADMA), two endogenous inhibitors of nitric oxide (NO) synthase [7]. Of note is that ADMA-NO pathway is involved in the development of fetal programming and programmed hypertension [8,9].

A maternal methyl-deficient diet was reported to alter components of the DNA methylation machinery during the early stages of organogenesis and cause metabolic disturbances in the offspring [10,11]. While most early reports showed that maternal methyl-donor diets have beneficial effects on offspring outcomes in several programming models [12,13], some subsequent studies demonstrated that these diets may induce hypermethylation and suppression of beneficial genes, leading to various diseases [4,14,15]. The kidney is a decisive organ for the regulation of BP and it is vulnerable to the nutritional insults of programming during nephrogenesis [16]. Renal programming has been identified as a driving mechanism of programmed hypertension [17]. Thus, in this study our first aim is to elucidate whether a maternal methyl-donor diet or a methyl-deficient diet affects renal transcriptome and ADMA-NO pathway to induce developmental programming of hypertension in adult offspring.

Several mechanisms are proposed to drive programming processes, leading to renal programming and programmed hypertension [1,18]. These mechanisms include oxidative stress, alteration of nutrient-sensing signals, and dysregulation of sodium transporters [1,8,9,17,18]. However, it is unclear whether these pathways are involved in the programmed hypertension induced by a low or high methyl-donor diet exposure.

Conversely, reprogramming, a strategy for reversing programming processes, is proposed by the DOHaD concept to shift the treatment from adulthood to early life [18]. Melatonin, a pineal hormone, has pleiotropic bioactivities and reprogramming effects [19,20,21]. Maternal melatonin therapy was reported to up-regulate a myriad of genes in the offspring kidney [20]. Given that melatonin has been reported to affect DNA methylation [21], and that maternal melatonin therapy prevented programmed hypertension via regulating ADMA-NO pathway [22], our second aim is to test whether maternal melatonin therapy can prevent programmed hypertension induced by a maternal methyl-donor diet via altering renal transcriptome, ADMA-NO pathway, and other above-mentioned mechanisms.

## 2. Materials and Methods 

### 2.1. Animal Study

This study was approved the Institutional Animal Care and Use Committee of the Kaohsiung Chang Gung Memorial Hospital (Permit Number: 2014111001) and performed under the Guidelines for the Care and Use of Laboratory Animals of the National Institutes of Health. Virgin Sprague Dawley (SD) rats, 12–16 weeks old, were obtained from BioLASCO (BioLASCO Taiwan Co., Ltd., Taipei, Taiwan). Rats were housed in a facility accredited by the Association for Assessment and Accreditation of Laboratory Animal Care International. Rats were exposed to a standard photoperiod of 12 h of light and 12 h of dark. The animals were mated on the basis of one male to one female. Females were examined daily during the mating period. The observation of vaginal plug was considered evidence of successful mating. Pregnant rats were randomly divided into four groups: controls, rats treated with a methyl-deficient diet (L-MD), rats treated with a high methyl-donor diet (H-MD), and rats treated with a high methyl-donor diet plus melatonin (H-MD+M) during the whole pregnancy and lactation period (i.e., a total of 6 weeks). The content of fat, protein, and carbohydrate in the normal diet represented 14.1%, 27.95%, and 58%, respectively of the total energy content of the diet as previously described [23]. A methyl-donor diet (C16083101, Research diet Inc., New Brunswick, NJ, USA) contained 5 g/kg diet of betaine (Sigma Aldrich, Saint Louis, MO, USA), 5.37 g/kg diet of choline (Sigma Aldrich, Saint Louis, MO, USA), 5.5 mg/kg diet of folic acid (Sigma Aldrich, Saint Louis, MO, USA) and 0.5 mg/kg diet vitamin B12 (Sigma Aldrich, Saint Louis, MO, USA) as previously described [24]. Methyl-deficient diet was a diet low in l-methionine (0.18%), devoid of choline and folic acid (517946, Dyets Inc., Bethlehem, PA, USA) [10]. Another group of methyl-donor diet-treated rats received 0.01% melatonin in drinking water during the entire pregnancy and lactation. The dose of melatonin was adopted based on our previous studies conducted in rats [20,22]. Melatonin was prepared twice weekly by dissolving the drug (10 mg) in 1 mL of 100% ethanol. This solution was then diluted with water to a final concentration of 0.01%. Water bottles were protected from light by wrapping in aluminum foil. After their birth, litters were culled to eight pups to standardize the received quantity of milk and maternal care. Body weight (BW) of the pups was not measured at birth to prevent maternal rejection. Because hypertension occurs at a higher rate and at an earlier age in males than females [25], only male offspring (*n* = 7–8 per group) were selected from each litter and used in subsequent experiments. 

BP was measured in conscious rats at 3, 4, 6, 8, 10, and 12 weeks of age by using an indirect tail-cuff method (BP-2000; Visitech Systems, Inc., Apex, NC, USA) [22]. To ensure accuracy and reproducibility, the rats were acclimated to restraint and tail-cuff inflation for 1 week before the experiment. Three stable measurements were obtained and were averaged. Male offspring were killed at 12 weeks of age. Heparinized blood samples were collected at the time of sacrifice. Kidneys were harvested after perfusion with phosphate-buffered saline (PBS), decapsulated, divided into cortex and medulla, flash-frozen in liquid nitrogen, and stored at −80 °C for further analysis. Plasma levels of NO_2_^−^ and NO_3_^−^ were measured by the Griess reaction, as previously described [26].

### 2.2. Quantitative Real-Time Polymerase Chain Reaction (qPCR)

RNA was extracted using TRI Reagent (Sigma, St. Louis, MO, USA) and treated with DNase I (Ambion, Austin, TX, USA) to remove DNA contamination. [23]. Two-step quantitative reverse transcription PCR (qPCR) was conducted using the Quantitect SYBR Green PCR kit (Qiagen, Valencia, CA, USA). DNA amplification and quantitation were performed in the iCycler iQ Multi-color Real-Time PCR Detection System (Bio-Rad, Hercules, CA, USA). Genes associated with nutrient-sensing signals were analyzed, including peroxisome proliferator-activated receptor (PPAR)-α (*Ppara*), -β (*Pparb*), and -γ (*Pparg*), PPARγ coactivator 1-α (PGC-1α encodes for *Pargc1ap*), sirtuin-1 (*Sirt1*), and protein kinase, AMP-activated, subunit-α2 (*Prkaa2*), -β2 (*Prkab2*), and -γ2 (*Prkag2*). Additionally, expression of four sodium transporters, namely, type 3 sodium hydrogen exchanger type 3 (*Slc9a3*), Na-K-2Cl cotransporter (*Slc12a1*), Na^+^/Cl^−^ cotransporter (*Slc12a3*), and Na^+^/K^+^-ATPase α1 subunit (*Atp1a1*), was analyzed. The 18S rRNA gene (*Rn18s*), a widely used normalization gene, was used as a reference. Primer sequences are provided in Table 1. All samples were run in duplicate. For the relative quantification of gene expression, the comparative threshold cycle (*C*_T_) method was employed. The averaged *C*_T_ was subtracted from the corresponding averaged *Rn18s* value for each sample, resulting in ∆*C*_T_. ∆∆*C*_T_ was achieved by subtracting the average control ∆*C*_T_ value from the average experimental ∆*C*_T_. The fold-change was established by calculating 2^−∆∆*C*T^ for experimental vs. reference samples. 

### 2.3. High-Performance Liquid Chromatography (HPLC)

The plasma levels of several components of the nitric oxide (NO) pathway, including l-arginine, l-citrulline, ADMA, and SDMA were measured using HPLC (HP series 1100; Agilent Technologies Inc., Santa Clara, CA, USA). O-phthalaldehyde/3-mercaptopropionic acid (OPA/3-MPA) was used as the derivative reagent [22]. Plasma homocysteine and creatinine levels were also measured using HPLC (HP series 1100, Agilent Technologies, Inc, Santa Clara, CA, USA) described previously [22,27].

### 2.4. Next-Generation Sequencing and Analysis

Kidney cortex samples (*n* = 3/group) were randomly chosen, pooled and used for next-generation sequencing (NGS) analysis (Welgene Biotech Co, Ltd., Taipei, Taiwan) as we described previously [20]. Purified RNA was quantified at 260 nm (OD600) by using ND-1000 spectrophotometer (Nanodrop Technology, Wilmington, DE, USA) and analyzed using a Bioanalyzer 2100 (Agilent Technology, Santa Clara, CA, USA) with RNA 6000 LabChip kit (Agilent Technologies, Santa Clara, CA, USA). We followed the Illumina protocol for library preparation and sequencing. Library construction was performed with the Solexa platform (Illumina Inc., San Diego, CA, USA). The sequence was directly determined using sequencing-by-synthesis technology via the TruSeq SBS Kit (Illumina, San Diego, CA, USA). Raw sequences were obtained with the Illumina Pipeline software CASAVA v1.8 (Illumina, San Diego, CA, USA), which was expected to generate 30 million reads per sample. Quantification for gene expression was calculated as reads per kilobase of exon per million mapped reads (RPKM) [28]. Our selection criteria to select differentially expressed genes (DEGs) include (1) minimum of a 2-fold difference in normalized read counts between groups, and (2) genes that changed by RPKM ≥ 0.3 in either control or L-MD group. *p* value was estimated for each gene and corrected for multiple testing (*q* value) by the Benjamini-Hochberg correction. The log2 fold change was used to partition the genes into up- and down-regulated groups. To find the related biological pathway, Gene Ontology analysis and Kyoto Encyclopedia of Genes and Genomes (KEGG) analysis were performed using the Database for Annotation, Visualization and Integrated Discovery (DAVID) v6.8 on line tool [29].

### 2.5. Immunohistochemistry Staining

Paraffin-embedded tissue was sectioned at 3 μm thickness. Tissue slides were deparaffinized with xylene and rehydrated in a series of ethanol solutions with decreasing concentrations. Following blocking with immunoblock (BIOTnA Biotech., Kaohsiung, Taiwan), the sections were incubated with an anti-8-hydroxydeoxyguanosine (8-OHdG) antibody (clone #N45.1, 1:100, JaICA, Shizuoka, Japan) at room temperature for 2 h, as previously described [22]. 8-OHdG is one of the most abundant oxidative products of cellular DNA damage. Immunoreactivity was revealed using the polymer-horseradish peroxidase (HRP) labeling kit (BIOTnA Biotech, Kaohsiung, Taiwan). For the substrate-chromogen reaction, 3,30-diaminobenzidine (DAB) was used. The sections were preserved under cover glass. Renal cells positive for 8-OHdG were examined in 10 randomly selected ×400 microscopic fields per section. The number of immunostained cells was expressed as a percentage [22].

### 2.6. Statistical Analysis 

Statistical analysis was conducted using one-way analysis of variance (ANOVA) with a Tukey post hoc test for multiple comparisons. BP was analyzed with two-way repeated-measures ANOVA, with a Tukey post hoc test. All values are given as mean ± standard error of the mean. A *p* < 0.05 was considered to be statistically significant. Analyses were performed with the use of the Statistical Package for the Social Sciences software (SPSS 14.0, SPSS, Chicago, IL, USA).

## 3. Results

### 3.1. Morphological Features, Biochemical Values, and Blood Pressure

Litter sizes were not significantly altered by L-MD, H-MD, or H-MD+M exposure of the maternal rat (pups per litter: control = 11 ± 0.4; L-MD = 10.3 ± 1; M-MD = 11 ± 0.9; and H-MD+M = 11 ± 1.2). As shown in Table 2, the mortality rate for each group was 0%. The body weight (BW) was lower in H-MD rats than in the L-MD group, while the reduction of BW was restored by melatonin treatment. Among the four groups, kidney weight was comparable and kidney weight-to-BW ratio was lowest in the H-MD+M group. At 12 weeks of age, systolic BP (SBP) significantly increased in L-MD and H-MD rats, but near normal in the group treated with melatonin. 

**Figure 1:**
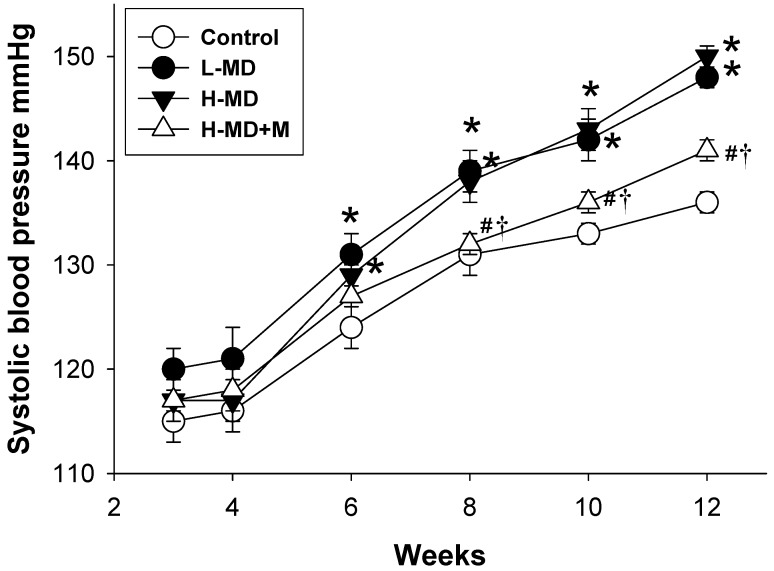
Effects of a maternal methyl-deficient diet (L-MD), high methyl-donor diet (H-MD), and high methyl-donor diet plus melatonin therapy (H-MD+M) on systolic blood pressure in male offspring. * *p* < 0.05 vs. control; ^#^ *p* < 0.05 vs. L-MD; ^†^ *p* < 0.05 vs. H-MD. *n* = 7–8/group.

As shown in Figure 1, the elevation of SBP in the L-MD and H-MD group was similar at six to 12 weeks of age. The SBP was significantly reduced by melatonin treatment at eight to 12 weeks of age, but not earlier. Blood creatinine level was not different among the four groups. 

### 3.2. Renal Transcriptome

**Figure 2:**
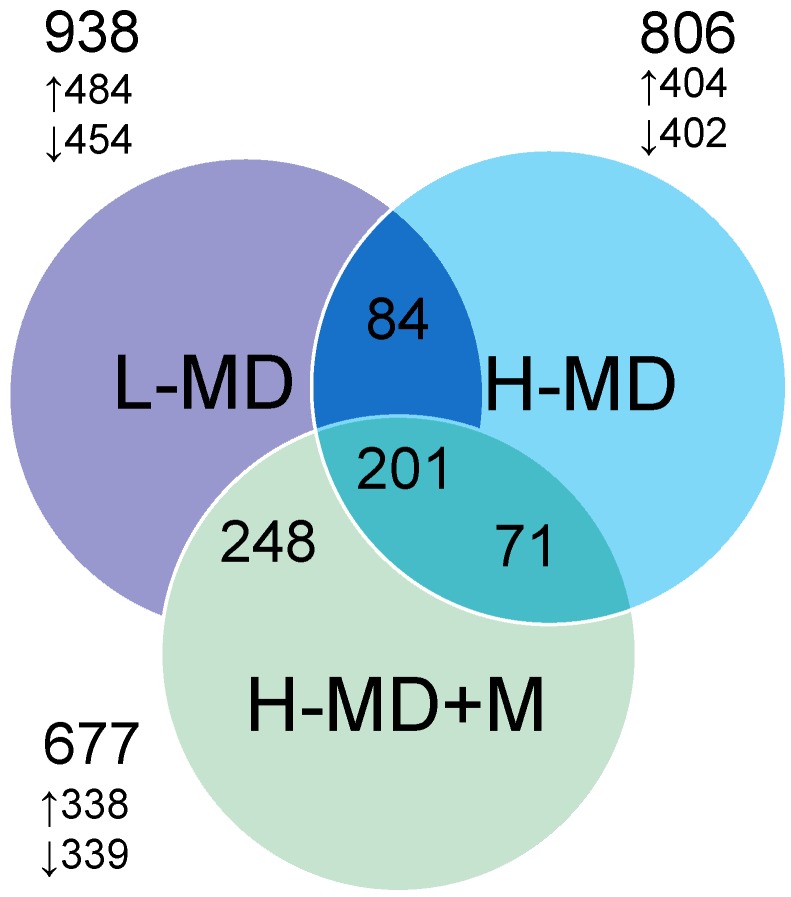
Venn diagram depicting unique and shared (overlapping circles) sets of differentially expressed genes (DEGs) in male offspring exposed to a methyl-deficient diet (L-MD, purple circle), high methyl-donor diet (H-MD, blue circle), and high methyl-donor diet plus melatonin therapy (H-MD+M, green circle). A total of 201 DEGs are shared by three groups. The total number, as well as number of up- or down-regulated genes identified during each group, is also indicated.

We next analyzed differential gene expression induced by L-MD, H-MD, and H-MD+M in the kidney. A total of 938 DEGs with 484 up-regulated and 454 down-regulated genes were identified between L-MD and control group. Next, there was a total of 806 DEGs (404 up- and 402 down-regulated genes) in the kidney between H-MD and control groups. Additionally, a total of 677 genes (338 up- and 339 down-regulated genes between H-MD+M vs. control) in 12-week-old offspring’s kidney Genes shared by different developmental windows are represented graphically by the Venn diagram shown in Figure 2. Among them, 201 shared genes were identified (Table S1). 

We next used DAVID v6.8 KEGG Pathway tool to identify biological processes from our gene lists. We found 10, 21, and 8 significantly related KEGG pathways in the kidney of the L-MD, H-MD, and H-MD+M group vs. control, respectively (Table 3). Our data showed that PPAR signaling pathway was a shared KEGG pathway, suggesting PPAR signaling might be a common pathway involved in the development of hypertension. PPAR signaling belongs to nutrient-sensing signals, which play a key role in fetal development. Maternal nutritional insults can affect these sensing signals during critical periods of fetal development that results in developmental programming of hypertension [30,31].

### 3.3. Nutrient-Sensing Pathway and Sodium Transporters

**Figure 3:**
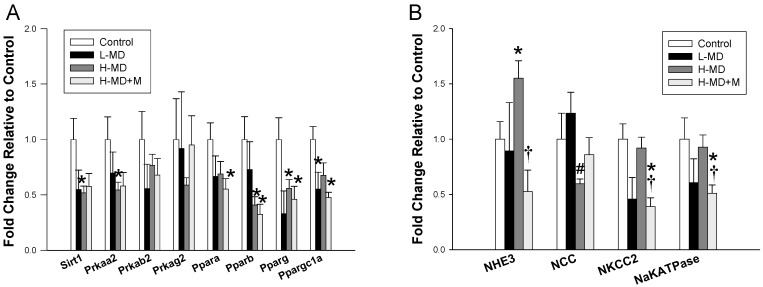
Effects of a maternal methyl-deficient diet (L-MD), high methyl-donor diet (H-MD), and high methyl-donor diet plus melatonin therapy (H-MD+M) on mRNA expression of (**A**) nutrient-sensing signals and (**B**) sodium transporters in male offspring kidneys at 12 weeks of age. * *p* < 0.05 vs. control; ^#^ *p* < 0.05 vs. L-MD; ^†^ *p* < 0.05 vs. H-MD. Data are mean ± SEM, *n* = 7–8/group.

We next analyzed genes associated with nutrient-sensing signals and sodium transporters. As shown in Figure 3, a methyl-deficient diet decreased renal *Ppargc1a* (coding for PGC-1α) expression. Renal mRNA expression of *Sirt1*, *Prkaa2* (coding for AMPKα2), *Pparb* (coding for PPARβ), *and Pparg* (coding for PPARγ) were lower in the L-MD group than those in controls. A methyl-donor diet combined with melatonin treatment caused a reduction of renal expression of *Ppara*, *Pparb*, *Pparg*, and *Ppargc1a* compared to controls. Additionally, a high methyl-donor diet significantly increased renal mRNA expression of *Slc9a3* (encoding for NHE3). Melatonin therapy reduced mRNA expression of *Slc9a3*, *Slc12a1* (encoding for NKCC2), and *Atp1a1* (encoding for NaKATPase) in the H-MD+M group compared with those in the H-MD group.

### 3.4. ADMA-NO Pathway

In order to explore the effects of a maternal methyl-deficient diet and a high methyl-donor diet on protein arginine methylation, we investigated the l-arginine-ADMA-NO pathway (Table 4). Plasma levels of l-citrulline, l-arginine, and ADMA were not different among the four groups. L-MD diet exposure increased plasma SDMA levels, while plasma SDMA level was lower in the H-MD+M rats compared to the L-MD group. In contrast, melatonin therapy significantly increased urinary NO_2_^−^ level in the H-MD+M group vs. the L-MD group. Additionally, the arginine methylation index, estimated by the ratio of the dimethylated arginine (ADMA+SDMA) to the unmethylated arginine, and homocysteine level were comparable among the four groups. Our data suggest dietary methyl nutrients and maternal melatonin therapy had a negligible effect on the protein arginine methylation in the adult offspring. Maternal melatonin therapy significantly increased urinary NO_2_^−^ level, a stable metabolite of NO, in H-MD+M rats compared to that in H-MD and L-MD group.

**Figure 4:**
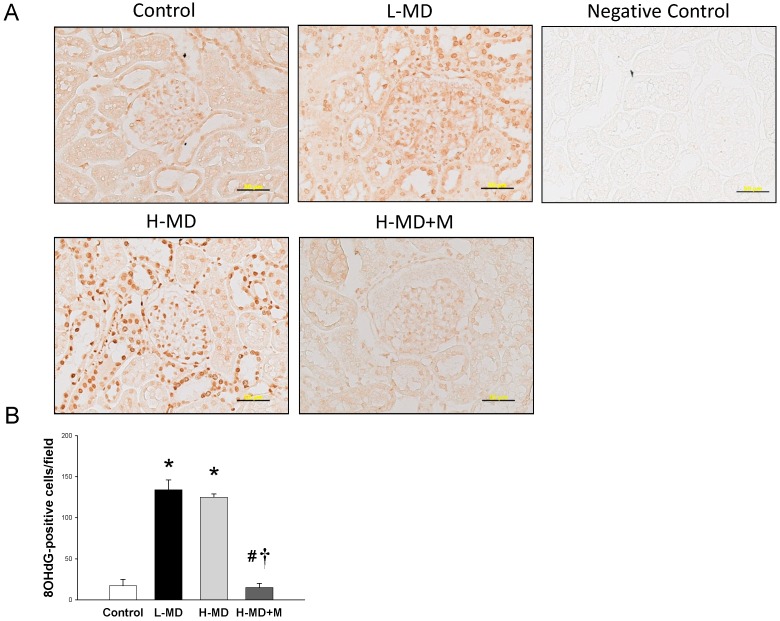
(**A**) Light micrographs illustrating immunostaining for 8-hydroxydeoxyguanosine (8-OHdG) in the kidney of 12-week-old male offspring exposed to a maternal methyl-deficient diet (L-MD), high methyl-donor diet (H-MD), and high methyl-donor diet plus melatonin therapy (H-MD+M). Bar = 50 μm. (**B**) Quantitative analysis of 8-OHdG-positive cells per microscopic field (×400). * *p* < 0.05 vs. control; ^#^ *p* < 0.05 vs. L-MD; ^†^ *p* < 0.05 vs. H-MD. *n* = 7–8/group.

We next evaluated 8-OHdG, a marker of oxidative DNA damage, in the kidney by using immunohistochemistry. Immunostaining of 8-OHdG in the glomeruli and renal tubules showed intense staining in the L-MD (134 ± 12 positive cells) and H-MD (125 ± 4 positive cells), while little staining in the H-MD+M (15 ± 5 positive cells) and controls (17 ± 8 positive cells) (Figure 4).

## 4. Discussion

Our work provides novel insight into the mechanisms by which maternal melatonin therapy protects adult male offspring against programmed hypertension induced by a high methyl-donor diet. The main findings in this study include: (1) exposure of the mothers to a methyl-deficient diet or a high methyl-donor diet resulted in programmed hypertension in their male offspring at 12 weeks of age; (2) maternal melatonin therapy attenuated high methyl-donor diet-induced programmed hypertension; (3) maternal methyl-deficient diet and high methyl-donor diet altered respectively 938 and 806 renal transcripts in adult offspring; (4) a total of 201 differentially expressed genes (DEGs) were shared by L-MD, H-MD, and H-MD+M exposure; and (5) the protective effects of melatonin on programmed hypertension is associated with reduced oxidative stress and plasma SDMA level, increased urinary NO_2_^−^ level, and reduced renal expression of sodium transporters.

To the best of our knowledge, this study is the first to show that exposure to both a methyl-deficient diet and a high methyl-donor diet during pregnancy and lactation cause elevation of BP in adult offspring. A methyl-deficient diet has been linked to the development of hypertension [32,33]. Here we report that reducing dietary methionine, choline, and folic acid from gestation throughout lactation leads to hypertension in adult offspring, which combines alterations of renal transcriptome and increased oxidative stress. On the other hand, a maternal high methyl-donor diet also caused programmed hypertension in adult offspring. Interestingly, a high methyl-donor diet did not lead to an increased global DNA methylation and gene silencing in adult offspring kidney. Regardless of whether mother rats were fed with a high or low methyl-donor diet, similar numbers of up- and down-regulated genes were altered in adult offspring’s renal transcriptome. Thus, regarding methylation status on the global level, both diets are likely to differentially affect beneficial and harmful genes, leading to programmed hypertension. Although a methyl-donor diet could be used for prevention of various human diseases [4,34], our data indicate that a maternal methyl-donor diet results in long-term alterations in renal transcriptome and programmed hypertension in adult offspring. In line with earlier reports demonstrating the beneficial effects of maternal melatonin therapy study in a number of models of developmental programming [19], we found that maternal melatonin therapy alleviates high methyl-donor diet-induced programmed hypertension.

A total of 201 DEGs were shared among three different groups vs. controls. However, none of them, to our knowledge, have shown a direct relationship with hypertension. We also found 10, 21, and 8 significantly related KEGG pathways in the kidney of the L-MD, H-MD, and H-MD+M group, respectively. However, whether these pathways might be involved in programmed hypertension remain unclear. Among them, we found that “complement and coagulation cascades” is a significant KEGG pathway shared by three groups. This is consistent with previous reports showing vast alterations in complement components and coagulation genes in two different programming models, one is a rat model of intrauterine growth retardation and the other is a maternal high-fructose diet model [35,36]. Another significant KEGG pathway is the PPAR signaling pathway, which was shared by the L-MD and H-MD+M group. Notably, previous reports have shown that early-life nutritional insults may program nutrient-sensing signals to regulate PPARs and their target genes, thereby causing programmed hypertension [37,38].

Our current study showed that a methyl-donor diet decreased mRNA expression of several nutrient-sensing signals, including *Sirt1*, *Prkaa2*, *Pparb*, *and Pparg*. Activation of SIRT/AMPK, PPARβ/δ, or PPARγ had antihypertensive effects [31,38,39]. Thus, signals formed by a methyl-donor diet appear in favor of hypertension. However, maternal melatonin therapy had a negligible effect on reprogramming nutrient-sensing signals. Additionally, a methyl-donor diet increased mRNA expression of sodium transporter NHE3 (*Slc9a3*). Since increased expression of sodium transporters trigger sodium retention and hypertension, our findings suggest that dysregulation of nutrient-sensing signals and sodium transporters in the kidneys may be the key mediators for the development programming of hypertension induced by a high methyl-donor diet. Of note is that maternal melatonin therapy prevented the H-MD-induced increase in *Slc9a3* mRNA level as well as decreased the *Slc12a1* and *Atp1a1* expression in offspring kidneys.

In addition to DNA methylation, the methyl-donor status is involved in arginine methylation. Like ADMA and SDMA, homocysteine is produced as a result of methylation reactions. Our data showed that arginine methylation index, represented as the (ADMA+SDMA)-to-l-arginine ratio, and homocysteine level in the plasma were not affected by maternal exposure to a methyl-deficient diet or a methyl-donor diet. In line with previous studies showing that melatonin increased NO to prevent the development of hypertension [19,22], we observed that maternal melatonin therapy reduced plasma SDMA level and increased urinary NO_2_^−^ level, an index of NO bioavailability, in the H-MD+M group. Additionally, we demonstrated the presence of oxidative stress damage, represented by a greater 8-OHdG staining in the kidneys of male offspring exposed to a high methyl-donor diet, which was prevented by maternal melatonin therapy. These findings support the notion that melatonin can be protective as a reprogramming intervention to restore the NO-ROS balance against programmed hypertension [18].

Emerging evidence documents reprogramming effects of melatonin treatment in different developmental programming animal models [18,19]. Melatonin has been reported to affect DNA methylation or act like a histone deacetylase (HDAC) inhibitor [21,40]. Consistent with our earlier report documenting that maternal melatonin therapy alters the expression of a myriad of genes in the offspring kidney [20], in the current study we showed for the first time that melatonin has a protective role on programmed hypertension induced by a methyl-donor supplemented maternal diet. 

A potential limitation of this study is the inability to test melatonin in the control and L-MD group. In pregnant rats, even at high doses up to 200 mg/kg/day, melatonin was reported to have no harmful effects on the development of rat pups [41]. We, hence, did not conduct a control+M group in the current study. However, further studies are warranted to elucidate whether melatonin has beneficial effects in the L-MD group, similar to those in the H-MD group. Second, we did not examine sex difference in response to L-MD as well as H-MD exposure. Since we measured BP in young adulthood and cardiovascular events occurred at an early age in males than females [25], only male rats were recruited in this study. Another limitation is that we did not use of several reference genes for validation of our qPCR results. Since no single gene is constitutively expressed in all tissues and under all experimental conditions, the expression stability of the intended references gene has to be verified as recommended by the MIQE guideline [42,43]. Last, we did not validate 201 shared DEGs identified by NGS RNA-Seq data using qPCR. Although we have previously shown that genes quantified by qPCR agreed with NGS data in other models of programmed hypertension [44], further studies are needed to validate these candidate genes in our NGS RNA-Seq data.

## 5. Conclusions

In conclusion, maternal melatonin therapy counterbalances the effects of high methyl-donor diet-induced programmed hypertension, primarily related to the reduction of oxidative stress, increased urinary NO_2_^−^ level, and decreased expression of sodium transporters in the kidney. Our NGS data indicated that pregnant rats exposed to a high methyl-donor diet caused long-term alterations of ~800 renal transcripts in their offspring. The nutrient-sensing signals and sodium transporters potentially were involved in the high methyl-donor diet-induced programmed hypertension. Conversely, the fact that a maternal methyl-deficient diet altered ~900 genes in the offspring’s renal transcriptome by which the adult BP can be modified. A better understanding of the mechanisms underlying DNA and protein methylation in the developmental programming of hypertension is crucial to aid in developing ideal methyl supplemented diet for improving fetal and offspring outcome. 

## Figures and Tables

**Table 1:** Quantitative real-time polymerase chain reaction primers sequences.

Gene	Forward	Reverse
*Sirt1*	5′-TGGAGCAGGTTGCAGGAATCCA-3′	5′-TGGCTTCATGATGGCAAGTGGC-3′
*Ppara*	5′-AGAAGTTGCAGGAGGGGATT-3′	5′-TTCTTGATGACCTGCACGAG-3′
*Pparrb*	5′-GATCAGCGTGCATGTGTTCT-3′	5′-CAGCAGTCCGTCTTTGTTGA-3′
*Pparg*	5′-CTTTATGGAGCCTAAGTTTGAGT-3′	5′-GTTGTCTTGGATGTCCTCG-3′
*Pargc1ap*	5′-CCCATTGAGGGCTGTGATCT-3′	5′-TCAGTGAAATGCCGGAGTCA-3′
*Prkaa2*	5′-AGCTCGCAGTGGCTTATCAT-3′	5′-GGGGCTGTCTGCTATGAGAG-3′
*Prkab2*	5′-CAGGGCCTTATGGTCAAGAA-3′	5′-CAGCGCATAGAGATGGTTCA-3′
*Prkag2*	5′-GTGTGGGAGAAGCTCTGAGG-3′	5′-AGACCACACCCAGAAGATGC-3′
*Slc9a3*	5′-CATTTGTCCCTTTCCGAATTG-3′	5′-CCAAATGGCAGCTCCAAATAG-3′
*Slc12a1*	5′-ACAGGAGGACCCATGACAAGA-3′	5′-GCAGCAGATACAGAGGCCACTA-3′
*Slc12a3*	5′-TGATCCGATGCATGCTCAA-3′	5′-CGCCTGCGCCGTAATC-3′
*Atp1a1*	5′-GGCTGTCATCTTCCTCATTGG-3′	5′-CGGTGGCCAGCAAACC-3′
*Rn18s*	5′-GCCGCGGTAATTCCAGCTCCA-3′	5′-CCCGCCCGCTCCCAAGATC-3′

**Table 2:** Summary of morphological values and blood pressure.

Parameter	Control	L-MD	H-MD	H-MD+M
	*n* = 8	*n* = 8	*n* = 7	*n* = 8
Body weight (BW) (g)	378 ± 9	405 ± 12	352 ± 9 ^b^	411 ± 7 ^c^
Left kidney weight (g)	1.39 ± 0.06	1.80 ± 0.08	1.60 ± 0.05	1.60 ± 0.05
Left kidney weight/ 100 g BW	0.49 ± 0.01	0.46 ± 0.01	0.46 ± 0.01	0.40 ± 0.01 ^a,b,c^
Systolic blood pressure (mmHg)	136 ± 1	150 ± 1 ^a^	148 ± 1 ^a^	141 ± 1 ^b,c^
Creatinine (μM)	17.6 ± 0.6	16.7 ± 0.7	19.8 ± 0.9	18.4 ± 0.5

^a^ *p* < 0.05 vs. control; ^b^ *p* < 0.05 vs. methyl-deficient diet (L-MD); ^c^ *p* < 0.05 vs. high methyl-donor diet (H-MD).

**Table 3:** Significantly regulated KEGG pathways in 12-week-old offspring’s kidney exposed to a methyl-deficient diet (L-MD), high methyl-donor diet (H-MD), and high methyl-donor diet plus melatonin treatment (H-MD+M).

Items	Count	%	*p*-Value	Benjamini
**L-MD: 10 KEGG pathways**				
Ribosome	23	4.4	4.3 × 10^−11^	9.1 × 10^−9^
Complement and coagulation cascades	9	1.7	2.3 × 10^−4^	2.4 × 10^−2^
Staphylococcus aureus infection	7	1.4	1.4 × 10^−3^	9.5 × 10^−2^
PPAR signaling pathway	7	1.4	8.4 × 10^−3^	3.6 × 10^−1^
African trypanosomiasis	5	1.0	1.1 × 10^−2^	3.7 × 10^−1^
Phagosome	11	2.1	1.5 × 10^−2^	4.1 × 10^−1^
Herpes simplex infection	11	2.1	2.8 × 10^−2^	5.7 × 10^−1^
Transcriptional misregulation in cancer	9	1.7	3.3 × 10^−2^	5.9 × 10^−1^
Malaria	5	1.0	4.6 × 10^−2^	6.7 × 10^−1^
Type I diabetes mellitus	5	1.0	9.7 × 10^−2^	8.9 × 10^−1^
**H-MD: 21 KEGG pathways**				
Complement and coagulation cascades	12	1.7	2.2 × 10^−5^	5.3 × 10^−3^
Choline metabolism in cancer	12	1.7	5.0 × 10^−4^	5.8 × 10^−2^
FoxO signaling pathway	11	1.6	1.5 × 10^−2^	7.0 × 10^−1^
Proteoglycans in cancer	14	2.0	1.7 × 10^−2^	6.3 × 10^−1^
Influenza A	12	1.7	2.7 × 10^−2^	7.3 × 10^−1^
Herpes simplex infection	14	2.0	2.8 × 10^−2^	6.7 × 10^−1^
TNF signaling pathway	9	1.3	2.9 × 10^−2^	6.3 × 10^−1^
Type II diabetes mellitus	6	0.9	2.9 × 10^−2^	5.8 × 10^−1^
Staphylococcus aureus infection	6	0.9	3.3 × 10^−2^	5.9 × 10^−1^
Type I diabetes mellitus	7	1.0	4.0 × 10^−2^	6.2 × 10^−1^
Arachidonic acid metabolism	7	1.0	5.2 × 10^−2^	6.9 × 10^−1^
Cytokine-cytokine receptor interaction	13	1.9	5.7 × 10^−2^	6.8 × 10^−1^
Maturity onset diabetes of the young	4	0.6	5.9 × 10^−2^	6.7 × 10^−1^
HTLV-I infection	16	2.3	6.1 × 10^−2^	6.6 × 10^−1^
Graft-versus-host disease	6	0.9	6.8 × 10^−2^	6.7 × 10^−1^
Pathways in cancer	20	2.9	7.1 × 10^−2^	6.7 × 10^−1^
Prostate cancer	7	1.0	7.2 × 10^−2^	6.5 × 10^−1^
Tuberculosis	11	1.6	8.2 × 10^−2^	6.8 × 10^−1^
ErbB signaling pathway	7	1.0	8.2 × 10^−2^	6.6 × 10^−1^
Allograft rejection	6	0.9	8.3 × 10^−2^	6.4 × 10^−1^
Bile secretion	6	0.9	8.7 × 10^−2^	6.4 × 10^−1^
**H-MD+M: 8 KEGG pathways**				
Complement and coagulation cascades	9	1.5	5.7 × 10^−4^	1.2 × 10^−1^
PPAR signaling pathway	7	1.2	1.6 × 10^−2^	8.2 × 10^−1^
Metabolic pathways	47	7.9	1.7 × 10^−2^	7.2 × 10^−1^
Bile secretion	6	1.0	3.9 × 10^−2^	8.8 × 10^−1^
Biosynthesis of unsaturated fatty acids	4	0.7	4.0 × 10^−2^	8.3 × 10^−1^
Staphylococcus aureus infection	5	0.8	5.3 × 10^−2^	8.6 × 10^−1^
TNF signaling pathway	7	1.2	6.8 × 10^−2^	8.8 × 10^−1^
Phagosome	10	1.7	7.6 × 10^−2^	8.8 × 10^−1^

**Table 4:** Plasma and urine levels of NO-related parameters.

Parameter	Control	L-MD	H-MD	H-MD+M
l-Citrulline (μM)	106 ± 7	110 ± 8	118 ± 14	108 ± 13
l-Arginine (μM)	341 ± 18	371 ± 32	404 ± 25	334 ± 20
ADMA (μM)	1.88 ± 0.26	1.8 ± 0.18	1.81 ± 0.19	1.56 ± 0.09
SDMA (μM)	3.01 ± 0.35	4.21 ± 0.17 ^a^	3.23 ± 0.37	2.89 ± 0.25 ^b^
(ADMA+SDMA)-to-l-arginine ratio (μM/μM)	0.014 ± 0.002	0.017 ± 0.001	0.012 ± 0.001	0.015 ± 0.002
Homocysteine (μM)	3.84 ± 0.41	3.67 ± 0.58	4.81 ± 0.48	3.13 ± 0.42
Urine NO_2_^−^ (μM/24 h/kg BW)	0.42 ± 0.07	0.32 ± 0.03	0.43 ± 0.15	0.55 ± 0.04 ^b^

^a^ *p* < 0.05 vs. control; ^b^ *p* < 0.05 vs. L-MD. Data are mean ± S.E.M., *n* = 7–8/group.

## Supplementary Materials

The following are available online at http://www.mdpi.com/2072-6643/10/10/1407/s1, Table S1: 201 shared genes in the kidney of offspring at 12 weeks of age exposed to a methyl-deficient diet (L-MD), high methyl-donor diet (H-MD), and high methyl-donor diet plus melatonin treatment (H-MD+M).
