# Supplementary material for: Maternal Melatonin Therapy Attenuates Methyl-Donor Diet-Induced Programmed Hypertension in Male Adult Rat Offspring
Source: Nutrients. 2018 Oct 2;10(10):1407. doi: 10.3390/nu10101407 (PMC6213858; doi:10.3390/nu10101407)
Supplement: Supplementary file 1 [file nutrients-10-01407-s001.pdf]

Supplementary Table S1 201 shared genes in the kidney of offspring at 12 weeks of age exposed to methyl-deficient diet (L-MD), high methyl-donor diet (H-MD), and high methyl-donor diet plus melatonin treatment (H-MD+M)

Gene ID	Gene Symbol
ENSRNOG00000000060	*Cplx1*
ENSRNOG00000000249	*Mettl23*
ENSRNOG00000000461	*Brd2*
ENSRNOG00000000827	*Ier3*
ENSRNOG00000000918	*Zbed5*
ENSRNOG00000000996	*Arpc1a*
ENSRNOG00000001009	*Bri3*
ENSRNOG00000001724	*Atp13a3*
ENSRNOG00000002280	*Sh3bgrl*
ENSRNOG00000002835	*Luc7l3*
ENSRNOG00000002874	*Rbm25*
ENSRNOG00000002911	*Alb*
ENSRNOG00000002946	*Socs3*
ENSRNOG00000003566	*Apoh*
ENSRNOG00000003581	*Pou2f1*
ENSRNOG00000003645	*Hnrnpab*
ENSRNOG00000003794	*Nmral1*
ENSRNOG00000003807	*Wnt9b*
ENSRNOG00000004239	*LOC100362999*
ENSRNOG00000004532	*Fam69b*
ENSRNOG00000006283	*LOC100362400*
ENSRNOG00000006636	*Otud6b*
ENSRNOG00000006759	*Ankrd24*
ENSRNOG00000006889	*Ambp*
ENSRNOG00000007081	*Xdh*
ENSRNOG00000007532	*RGD1305178*
ENSRNOG00000008757	*Tmem218*
ENSRNOG00000009086	*Apcs*
ENSRNOG00000009139	*Fubp3*
ENSRNOG00000009163	*Fam133b*
ENSRNOG00000009382	*Mettl8*
ENSRNOG00000009530	*RGD1561252*
ENSRNOG00000009897	*Clcnkb*
ENSRNOG00000010019	*Fam166a*

ENSRNOG00000010181	*Clec4d*
ENSRNOG00000010262	*Hdc*
ENSRNOG00000010478	*LOC299282*
ENSRNOG00000010489	*Samd4a*
ENSRNOG00000011096	*LOC100912369*
ENSRNOG00000011351	*Mat1a*
ENSRNOG00000011542	*Apopt1*
ENSRNOG00000011799	*Ccdc159*
ENSRNOG00000011827	*AABR07009397.1*
ENSRNOG00000011907	*Krt23*
ENSRNOG00000011918	*Vsx2*
ENSRNOG00000012677	*AABR07065598.1*
ENSRNOG00000012752	*Cklf*
ENSRNOG00000013062	*Cyp24a1*
ENSRNOG00000013118	*Atox1*
ENSRNOG00000013238	*LOC103693210*
ENSRNOG00000013301	*Bean1*
ENSRNOG00000014117	*Hmox1*
ENSRNOG00000014362	*Nudt14*
ENSRNOG00000014387	*Chac1*
ENSRNOG00000014414	*Qrfpr*
ENSRNOG00000014739	*Wfdc2*
ENSRNOG00000014846	*Anp32a*
ENSRNOG00000014861	*Rnf183*
ENSRNOG00000014964	*Hp*
ENSRNOG00000015156	*Gal*
ENSRNOG00000015772	*AC097890.1*
ENSRNOG00000015844	*Snrpd2*
ENSRNOG00000015880	*Dpep1*
ENSRNOG00000016058	*Kazald1*
ENSRNOG00000016275	*Ttr*
ENSRNOG00000016580	*Rps23*
ENSRNOG00000017054	*Kcne3*
ENSRNOG00000017223	*Plg*
ENSRNOG00000017243	*Bnip3*
ENSRNOG00000017381	*Itih4*
ENSRNOG00000017383	*l7Rn6*
ENSRNOG00000017463	*Bloc1s3*

ENSRNOG00000017571	*Ndufa2*
ENSRNOG00000017689	*Itih3*
ENSRNOG00000018059	*Ihh*
ENSRNOG00000018477	*Otud4*
ENSRNOG00000018610	*Pde6d*
ENSRNOG00000018680	*Rpl17*
ENSRNOG00000018711	*Ppcdc*
ENSRNOG00000018904	*Dtymk*
ENSRNOG00000018935	*AABR07001497.1*
ENSRNOG00000018939	*Rexo2*
ENSRNOG00000019442	*Josd2*
ENSRNOG00000019864	*Fam132a*
ENSRNOG00000021010	*Arl2*
ENSRNOG00000021029	*Hamp*
ENSRNOG00000021960	*AC109958.1*
ENSRNOG00000022502	*Nsrp1*
ENSRNOG00000022953	*Ccdc163*
ENSRNOG00000023320	*Tspan1*
ENSRNOG00000023762	*Rsrc1*
ENSRNOG00000025074	*Fgg*
ENSRNOG00000025810	*Grcc10*
ENSRNOG00000025909	*Uqcc2*
ENSRNOG00000026067	*Wfdc10a*
ENSRNOG00000026519	*AABR07044711.1*
ENSRNOG00000026594	*LOC100909776*
ENSRNOG00000026813	*AABR07044933.1*
ENSRNOG00000027849	*Wipf2*
ENSRNOG00000028083	*Zmym6nb*
ENSRNOG00000028330	*AABR07067600.1*
ENSRNOG00000028582	*LOC688389*
ENSRNOG00000028690	*LOC100362366*
ENSRNOG00000028707	*Defa7*
ENSRNOG00000029115	*RGD1564883*
ENSRNOG00000029386	*RT1-N2*
ENSRNOG00000029627	*LOC100362684*
ENSRNOG00000030058	*LOC100359600*
ENSRNOG00000030174	*Gm18025*
ENSRNOG00000030387	*Kng1l1*

ENSRNOG00000030938	*RGD1564074*
ENSRNOG00000031167	*Srxn1*
ENSRNOG00000031207	*LOC500035*
ENSRNOG00000031211	*Acsm5*
ENSRNOG00000031579	*LOC100363469*
ENSRNOG00000031591	*AABR07032255.2*
ENSRNOG00000031834	*Nkain4*
ENSRNOG00000032327	*Pdia5*
ENSRNOG00000032596	*RT1-T24-1*
ENSRNOG00000032669	*Serpina1*
ENSRNOG00000032740	*Tmem258b*
ENSRNOG00000033153	*Sfn*
ENSRNOG00000033517	*LOC100360791*
ENSRNOG00000033619	*Apof*
ENSRNOG00000034161	*Cox6b1*
ENSRNOG00000037673	*RGD1565183*
ENSRNOG00000037897	*LOC100910336*
ENSRNOG00000037923	*Dmrtc1c1*
ENSRNOG00000038370	*Ahsg*
ENSRNOG00000038375	*AABR07026311.1*
ENSRNOG00000038999	*RT1-A1*
ENSRNOG00000039850	*Pigp*
ENSRNOG00000040350	*Mir675*
ENSRNOG00000042344	*Smim22*
ENSRNOG00000042547	*RGD1565566*
ENSRNOG00000042690	*Zmat4*
ENSRNOG00000045686	*Nfs1*
ENSRNOG00000045963	*LOC100360654*
ENSRNOG00000045967	*AABR07064061.1*
ENSRNOG00000046094	*Cd151*
ENSRNOG00000046271	*LOC100911685*
ENSRNOG00000046450	*LOC102550729*
ENSRNOG00000046535	*Ppm1m*
ENSRNOG00000046834	*C3*
ENSRNOG00000047915	*AABR07051550.1*
ENSRNOG00000048478	*Kcne4*
ENSRNOG00000049179	*Pidd1*
ENSRNOG00000049198	*LOC684841*

ENSRNOG00000049308	*Sfxn2*
ENSRNOG00000049425	*Fam167b*
ENSRNOG00000049489	*LOC102552166*
ENSRNOG00000050061	*Mcart1*
ENSRNOG00000050552	*Snap23*
ENSRNOG00000050828	*Vkorc1*
ENSRNOG00000051181	*Rn50_X_0743.2*
ENSRNOG00000051346	*Rn50_X_0635.3*
ENSRNOG00000051482	*Hmg1l1*
ENSRNOG00000051483	*Sepw1*
ENSRNOG00000051733	*AABR07010502.1*
ENSRNOG00000051880	*AABR07021996.1*
ENSRNOG00000052119	*AABR07067556.1*
ENSRNOG00000052122	*Rn50_20_0056.5*
ENSRNOG00000052141	*Rps10l1*
ENSRNOG00000053437	*AC103090.1*
ENSRNOG00000053452	*LOC100361457*
ENSRNOG00000053891	*Phf11b*
ENSRNOG00000054251	*Clec7a*
ENSRNOG00000055020	*LOC679731*
ENSRNOG00000055025	*Fam25a*
ENSRNOG00000055050	*AC095267.2*
ENSRNOG00000055067	*5S_rRNA*
ENSRNOG00000055068	*Ppial4d*
ENSRNOG00000055160	*AABR07033249.1*
ENSRNOG00000056153	*Fam46b*
ENSRNOG00000056174	*Pdzd4*
ENSRNOG00000056342	*U1*
ENSRNOG00000056393	*AABR07051555.1*
ENSRNOG00000056765	*Rpl10*
ENSRNOG00000056767	*Potea*
ENSRNOG00000057054	*AABR07028907.1*
ENSRNOG00000057527	*RNase_MRP*
ENSRNOG00000057580	*AABR07049866.1*
ENSRNOG00000057753	*Nup62cl*
ENSRNOG00000058050	*Mtfr2*
ENSRNOG00000058389	*AABR07006475.1*
ENSRNOG00000058408	*AC108588.1*

ENSRNOG00000058589	*AABR07046778.1*
ENSRNOG00000059110	*AABR07068341.1*
ENSRNOG00000059773	*AABR07051218.1*
ENSRNOG00000059883	*AABR07052758.1*
ENSRNOG00000059911	*Hgsnat*
ENSRNOG00000059957	*AABR07021734.2*
ENSRNOG00000060187	*AABR07053516.2*
ENSRNOG00000060288	*LOC100365062*
ENSRNOG00000061056	*AABR07042514.3*
ENSRNOG00000061212	*AABR07071374.1*
ENSRNOG00000061752	*AABR07021199.1*
ENSRNOG00000061816	*AABR07027870.1*
ENSRNOG00000061821	*AC109891.1*
ENSRNOG00000061844	*AABR07005618.1*
ENSRNOG00000061943	*Rn60_7_1214.2*
